# Assessment of retinal and choroidal microcirculation after unilateral recession–resection surgery for horizontal strabismus by swept-source optical coherence tomography angiography

**DOI:** 10.1038/s41598-023-46670-3

**Published:** 2023-11-07

**Authors:** Yang Meng, Yishuang Xu, Di Xiao, Changzheng Chen, Dihao Hua, Yonghong Xu

**Affiliations:** Department of Ophthalmology, Renmin Hospital of Wuhan University, Wuhan, 430060 China

**Keywords:** Diseases, Health care, Medical research

## Abstract

This study explored the possible hemodynamic changes of the retina and choroid after horizontal strabismus surgery using swept-source optical coherence tomography angiography (SS-OCTA). 32 eyes of 32 patients who underwent unilateral horizontal rectus muscle recession–resection surgery were included. SS-OCTA examinations were performed preoperatively and one week postoperatively. Several OCTA measurements were used, including vessel density (VD) of the superficial vascular complex (SVC), VD of the deep vascular complex (DVC), VD of the choriocapillaris (CC), choroidal vascular index (CVI) and choroidal thickness (CT). No significant change in VD of SVC, DVC, and CC was observed whereas CT increased significantly with CVI unchanged. Recession–resection surgery for horizontal strabismus seemed not to significantly influence the microcirculation of the retina and CC in the early postoperative period. However, choroidal thickening happened with a constant CVI probably due to the postoperative inflammation. Further studies are needed to investigate the long-term effects of unilateral recession–resection surgery for horizontal strabismus on the microcirculation of the retina and choroid.

## Introduction

Strabismus, also known as squint, is the misalignment of the visual axes^1^. It is a commonly encountered ocular disease worldwide, with the prevalence ranging from 0.8 to 5.65% in children and 1.0–4.0% in adults, respectively^2–7^. If left untreated, strabismus will only affect the cosmetic appearance of patients but also detriment their visual functions such as binocular vision and stereopsis^5^. In addition, studies have shown that strabismus has a negative impact on patient’s quality of life, which can be improved by appropriate surgical interventions^8^.

The potential changes in ocular blood flow after strabismus surgery have always been captivating ophthalmologists. The anterior segment of the eye is supplied by the anterior ciliary artery and long posterior ciliary artery, of which the former accounts for 70–80% of the whole blood supply^9^. During strabismus surgery, anterior ciliary arteries are transected, thus it is speculated that hemodynamic changes may happen to the eye after surgery^10^. Early work attempted to the potential hemodynamic changes via different approaches, e.g., indocyanine green angiography, color fundus photography, and color Doppler ultrasonography^10–12^. However, in view of the inconsistent conclusions drawn by previous studies, it remains obscure whether ocular blood flow will change significantly following strabismus surgery^12–14^.

The advent of swept-source optical coherence tomography angiography (SS-OCTA) has made it possible to visualize and assess the retinal and choroidal structure and vasculature in a rapid and non-invasive way^15^. To date, only a few recent studies have investigated the microvascular changes of the retina and choriocapillaris after strabismus surgery and made conflicting conclusions^9, 16^. Besides, to our knowledge, no existing study has investigated the changes in choroidal thickness (CT) and choroidal vascular index (CVI) using SS-OCTA.

Therefore, the purpose of this study is to use advanced SS-OCTA to evaluate the possible hemodynamic changes of the retina and choroid after unilateral recession–resection surgery for horizontal strabismus.

## Materials and methods

This is a prospective observational study. This study was conducted in accordance with tenets of the Declaration of Helsinki and was approved by the Ethical Review Committee of Renmin Hospital of Wuhan University. Informed consent was obtained for all participants before recruitment. For participants of 18 years or older, the informed consent was signed by themselves, and for those under 18 years, the informed consent was signed by their parents or guardians.

Participants who underwent unilateral horizontal rectus muscle surgery were enrolled at Renmin Hospital of Wuhan University from May 2022 to October 2022. All the diagnosis was made by an experienced specialist (Y.H.X.) after performing comprehensive examinations on all patients. The examinations included intraocular pressure, ocular motility, mydriatic optometry under cycloplegia, axial length, slit-lamp biomicroscopy anterior segment examination, dilated fundus examination, and strabismus tests (cover-uncover test, prism and alternate cover test, and Krimsky test). Radiological examinations (cranial CT and MRI) were performed when necessary. All patients were horizontal strabismus and had received unilateral recession–resection surgery. All strabismus operations were performed by an experienced expert (Y.H.X.) following standard procedures. Patients wouldbe excluded if they had: (1) other ocular diseases such as intraocular inflammation and heritable ocular diseases; (2) ocular hypertension with intraocular pressure > 21 mmHg; (3) a history of strabismus surgery or intraocular surgery; (4) refractive error < − 3 dioptres; (5) ocular media opacification that might affect the accurate measurement of OCTA; (6) any systemic disease that could affect the eye such as diabetes and hypertension; (7) a history of smoking, alcohol abuse, or drug problems; and (8) OCTA images with signal strength less than 7/10.

SS-OCTA examinations were conducted preoperatively and one week after surgery using the VG100 system (SVision Imaging, Ltd., Luoyang, China) by two trained examiners (Y.M. and Y.S.X.). Each participant was asked to avoid having cola, coffee, or any other caffeine-containing drink within 24 h before the examination. All participants were required to have a rest for 10 min before the OCTA examination. OCTA scans were conducted between 1:00 p.m. and 6:00 p.m. to minimize the influence of diurnal variation of the choroidal structure^17^.

The SS-OCTA device had a scan rate of 100,000 A-scans per second and a central wavelength of approximately 1050 nm. OCTA examinations were performed using the three-dimensional scan protocol with a 6 mm×6 mm scan area (1024 × 1024 B-scans) centered on the fovea. Built-in eye-tracking software was used to reduce eye motion artifacts. The segmentation of the retinal and choroidal layers was achieved using a validated built-in segmentation algorithm and was then carefully checked and calibrated by two experienced clinicians (Y.H.X. and D.H.H.).

**Figure 1:**
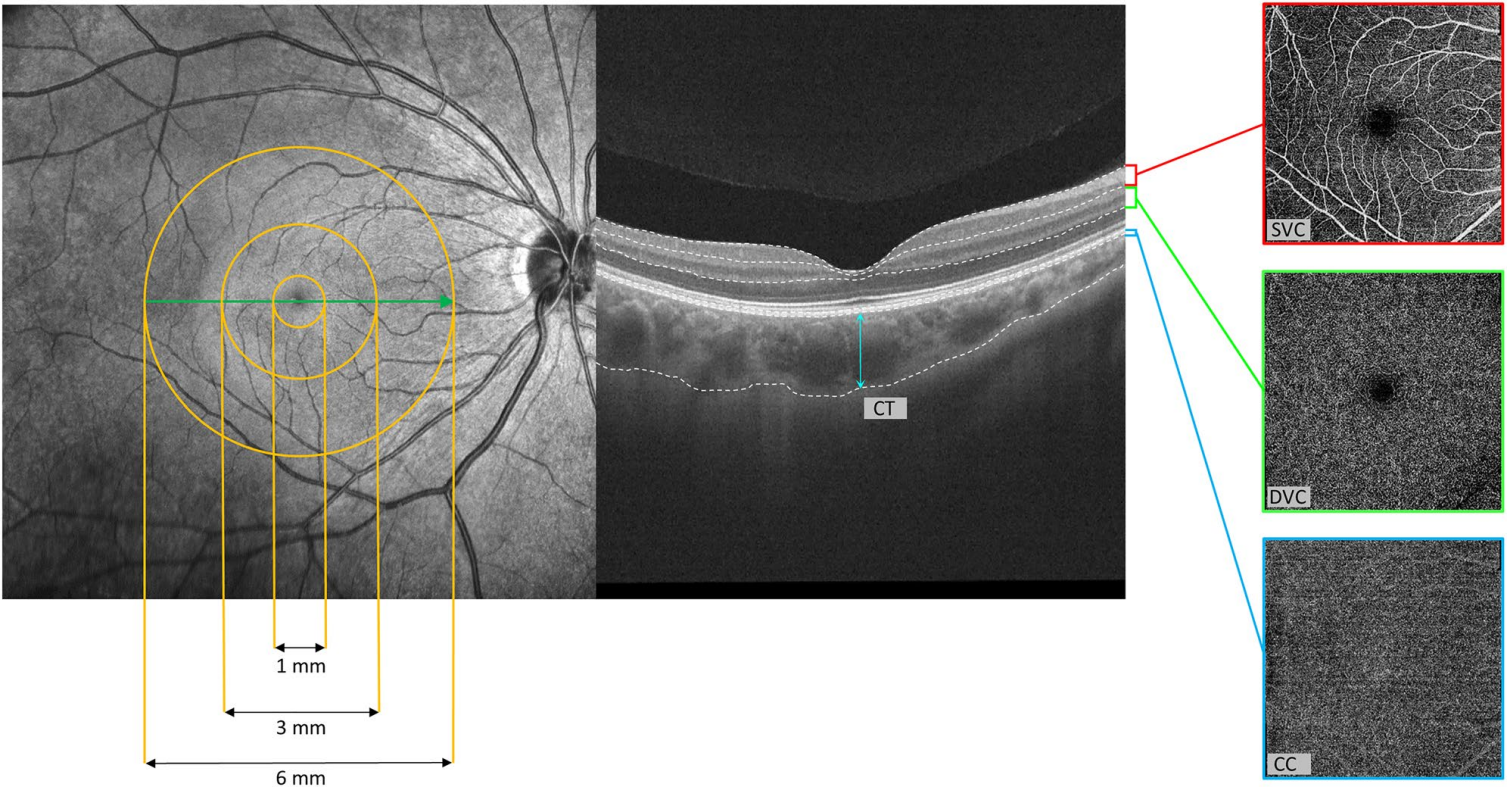
Segmentation of the macular subregions, structure, and microvasculature. *SVC* superficial vascular complex, *DVC* deep vascular complex, *CC* choriocapillaris, *CT* choroidal thickness.

The macula was stratified into three subregions as follows: the foveal region defined by a circle with a diameter of 1 mm centered at the foveal center, the parafoveal region defined by the annular area between 2 circles with diameters of 1 and 3 mm, and the perifoveal region defined by the annular area between 2 circles with diameters of 3 and 6 mm (Fig. 1). For this study, the following OCTA measurements were used: vessel density (VD) of the superficial vascular complex (SVC), VD of the deep vascular complex (DVC), VD of the choriocapillaris (CC), CVI and CT (Fig. 1). The VD was defined as the percentage of the area occupied by the projection of blood vessels in a certain retinal projection image. The SVC referred to the microvasculature from the internal limiting membrane (ILM) to the junction of the inner plexiform layer (IPL) and inner nuclear layer (INL), whereas DVC referred to the microvasculature from the IPL/INL junction to 25 µm below the outer plexiform layer (OPL). The CC was automatically segmented from the Bruch’s membrane to 25 μm below by the built-in AI algorithm. In this study, "CVI" specifically refers to three-dimensional (3D) CVI. The CVI was calculated built-in algorithm as the ratio of the choroidal vascular volume to the total choroidal volume. First, the OCTA device performed intensive B-scans (1024 × 1024) of the entire macular region (6 mm × 6 mm). The luminal area (LA) and total choroidal area (TCA) in each B-scan image were calculated, and then the LA and TCA were converted to the corresponding volumes by multiplying the distance between each two successive B-scans. Eventually, all the volumes were added up to the choroidal vascular volume and total choroidal volume. Since the CC is a thin monolayer only accounting for a very small part of the total choroidal volume, the CVI mainly reflects the vascularity of the layers with larger blood vessels (namely the Haller's layer and Sattler's layer)^18, 19^. The CT was defined as the distance between the Bruch’s membrane and the choroid-sclera interface (Fig. 1).

All analyses were implemented by IBM SPSS Statistics for Windows (Version 26.0. Armonk, NY: IBM Corp). Shapiro-Wilk test was used to detect the normality of all variables. Since all the studied variables were continuous variables, the paired t-test was used for variables of normal distribution whereas the Wilcoxon signed-rank test was used for those of non-normal distribution. All statistical analyses were performed two-tailed and p < 0.05 was considered statistically significant.

### Institutional Review Board Statement

The study was conducted in accordance with the Declaration of Helsinki, and approved by the Ethical Review Committee of Renmin Hospital of Wuhan University.

### Informed consent

Informed consent was obtained from all subjects involved in the study.

## Results

A total of 32 eyes of 32 patients were included in this study, among which 19 (59.4%) were female. The average age of the participants was 27.9 ± 11.8 years. All patients were horizontal strabismus (exotropia or endotropia). All patients had unilateral horizontal rectus muscle surgery (recession–resection).

Table 1 showed the preoperative and postoperative measurements of retinal vascular density at the level of SVC and DVC in all the macular subregions. No significant difference was observed between the preoperative and postoperative measurements of VD of SVC and DVC in all the subregions (all p > 0.05).

**Table 1:** Measurements of the superficial vascular complex and deep vascular complex.

Vascular density (%)	Zone	Preoperative (Mean ± SD)	Postoperative (Mean ± SD)	P value
SVC	Foveal	10.42 ± 5.04	10.61 ± 4.55	0.360*
	Parafoveal	49.89 ± 8.58	49.24 ± 8.24	0.199
	Perifoveal	51.50 ± 7.34	50.38 ± 7.38	0.053
DVC	Foveal	22.55 ± 6.86	21.99 ± 6.97	0.258
	Parafoveal	56.07 ± 3.24	55.52 ± 3.76	0.261
	Perifoveal	53.73 ± 4.28	54.03 ± 3.31	0.654*

*SVC* superficial vascular complex, *DVC* deep vascular complex.

*Wilcoxon signed-rank test. Others: paired sample t-test.

Table 2 demonstrated the preoperative and postoperative choroidal measurements. No significant difference was detected between the preoperative and postoperative VD of CC values and CVI values in any of the macular subregions (all p > 0.05). However, the CT in all the subregions increased significantly after strabismus surgery (all p < 0.001).

**Table 2:** Measurement of choroidal parameters.

Variables	Zone	Preoperative (Mean ± SD)	Postoperative (Mean ± SD)	P value
VD of CC (%)	Foveal	69.81 ± 8.75	71.23 ± 7.76	0.214
	Parafoveal	68.33 ± 7.66	68.74 ± 5.19	0.578
	Perifoveal	68.77 ± 5.31	69.45 ± 3.72	0.400*
CVI	Foveal	0.39 ± 0.10	0.40 ± 0.09	0.652
	Parafoveal	0.38 ± 0.09	0.39 ± 0.08	0.262*
	Perifoveal	0.36 ± 0.07	0.36 ± 0.07	0.341
CT (μm)	Foveal	302.6 ± 99.5	324.2 ± 103.5	** < 0.001**
	Parafoveal	299.6 ± 95.1	319.6 ± 98.4	** < 0.001**
	Perifoveal	291.4 ± 84.3	311.4 ± 89.7	** < 0.001**

*VD* vascular density, *CC* choriocapillaris, *CVI* choroidal vascular index, *CT* choroidal thickness.

*Wilcoxon signed-rank test. Others: paired sample t-test.

Bolded P values are of statistical significance.

## Discussion

In this study, we used swept-source OCTA to evaluate the potential hemodynamic changes of the retina and choroid in patients who underwent strabismus surgery. We observed no significant changes in the hemodynamic parameters of the retina (VD of SVC and DVC), or choroid (CVI and VD of CC) in any examined macular subregions. However, CT increased significantly in all the subregions one week following surgery.

Earlier researchers have tried to investigate the impact of strabismus surgery on ocular hemodynamics in different ways. Based on color Doppler imaging, some studies have investigated the hemodynamic changes in the retrobulbar blood flow following strabismus surgery and obtained conflicting conclusions^13, 14, 20^. Lee et al. found that strabismus surgery increased the blood flow of the ophthalmic artery (OA) on postoperative day 1 but the blood flow went back to baseline level one week after surgery and remained stable at one month^13^. Nevertheless, Akyüz et al. suggested that strabismus surgery did not have a measurable effect on the retrobulbar blood flow at both postoperative day 1 and day 30^14^. However, it should be noted that orbital color Doppler imaging has poor sensitivity when measuring vascular parameters and the accuracy and stability highly depend on the experience of the examiners. Also, color Doppler imaging can only indirectly infer the blood flow of larger vessels, e.g., the OA and central retinal artery (CRA), through parameters such as peak systolic blood flow velocity and resistivity index, thus being unable to well detect the retinal and choroidal microcirculation. Zhou et al. used a computer-assisted quantitative assessment software to assess the retinal vessel caliber changes on color fundus photographs^10^. They found the mean CRA equivalent increased significantly on postoperative day 1 but returned to baseline level at one week. However, the change of CRA equivalent might not directly represent the change of retinal microcirculation, and the use of routinely taken color fundus photographs to assess the retinal vascular diameters overlooked the systolic or diastolic effect on retinal vascular diameter.

OCTA has several advantages over the use of color Doppler imaging or color fundus photographs for studying hemodynamics and structure alterations after strabismus surgery. Based on motion signal differences in successive B-scans at the same location, OCTA provided us with a fast and non-invasive imaging technology to evaluate the retinal and choroidal structures and microcirculation^21^. Besides, the high resolution in the axial direction of OCTA can help analyze the microcirculation of the retina and choroid in different layers, such as the SVC and DVC of the retina as well as CC and CVI of the choroid^21^. Moreover, OCTA parameters had good repeatability for both healthy and diseased individuals^22, 23^. To our knowledge, only a few studies have investigated the potential hemodynamic changes of the macula after strabismus surgery^9, 16, 24^. Inal et al. evaluated the retinal vascular diameter changes preoperatively and 3 months postoperatively^24^. They found a statistically significant increase in VD of both the superficial and deep capillary plexus postoperatively. Çelik and colleagues observed no significant changes in retinal hemodynamics one week and one month postoperatively but detected a transient increase of the VD of CC at one week^9^. In a recent study, Vagge et al. assessed the macular blood flow on the first day postoperatively and one month postoperatively^16^. They showed a transitory increase of blood flow in the deep capillary plexus and CC only atday 1 following surgery. Nonetheless, CVI and CT were not investigated in these studies. Besides, our study design was more stringent. We ruled out factors that might affect the chorioretinal microcirculation and choroid thickness as much as possible, such as systemic disease, smoking, drug or alcohol abuse, caffeine-containing products, physical exercise, and diurnal variation of the choroid^17, 25–27^.

Our study suggests that recession–resection surgery for horizontal strabismus will not significantly impact the microcirculation of the retina and CC in the early postoperative period. However, choroidal thickening was found at the foveal, parafoveal, and perifoveal regions whereas the CVI remained constant. Since CVI is defined as:

1 $${\text{CVI }} = \frac{CVV}{{V_{total} }} = \frac{CVV}{{CVV + CSV}}$$

where V_total_ is the total choroidal volume, CVV is the choroid vessel volume, and CSV is the choroidal stroma volume. And the increase in CT represented the increase of V_total_. According to Eq. (1), only when the CVV and V_total_ increased at the same time could CVI remain constant. It can be further inferred that the CSV has also increased, otherwise, CVI would not remain unchanged. A possible explanation for this phenomenon is periocular inflammation. Choroidal thickening after cataract surgery has been widely reported and postoperative inflammation was considered an important cause^28–30^. Besides, many inflammatory disorders have been known to lead to choroidal thickening^31, 32^. Thus, it is likely that the increase in CT following strabismus surgery resulted from postoperative inflammation. Suture materials used in strabismus surgery were reported to cause postoperative inflammation^33–35^. Besides, surgical procedures would inevitably cause damage to blood vessels and muscles, resulting in a series of reactions such as the secretion of proinflammatory cytokines and activation of neutrophils^36, 37^. Then vasodilatation, increase of vascular permeability, and fluid exudation will happen via a complex immune cascade^36, 38^. The dilation of choroid vessels would explain the increase of CVV whereas the higher vascular permeability could contribute to an elevated fluid exudation from vessels into the stroma, causing an increased CSV. The simultaneous rise of CSV and CVV resulted in the increased CT and constant CVI observed in this study, although further research is needed to verify this hypothesis.

Our study has several limitations. First, this is a single-center study with a limited number of patients, thus further studies of larger sample sizes are needed. Another limitation is that we evaluated the retinal and choroidal microcirculation only one week after surgery thus the long-term effect of recession–resection surgery for horizontal strabismus on the retina and choroid needs further study. Finally, since we included patients with horizontal strabismus (esotropia and exotropia), possible differences between these two types of horizontal strabismus may have been overlooked, which would be an interesting topic for future exploratory work. While we acknowledge these limitations, our results may offer some new insights into the dispute over whether chorioretinal changes exist after strabismus surgery.

## Conclusions

In summary, we showed that recession–resection surgery for horizontal strabismus did not significantly impact the microcirculation of the retina and CC in the early postoperative period. However, choroidal thickening happened in the case of a constant CVI which is likely due to postoperative inflammation. Further studies with a longer follow-up are needed to investigate the long-term effects of recession–resection strabismus surgery on the retina and choroid.

## Data Availability

The datasets generated during and/or analyzed during the current study are not publicly available but are available from the corresponding author on reasonable request.
